# CRISPR-Cas9 mediated generation of a conditional poly(A) binding protein nuclear 1 (*Pabpn1*) mouse model reveals an essential role for hematopoietic stem cells

**DOI:** 10.1038/s41598-022-11203-x

**Published:** 2022-05-03

**Authors:** Pia Sommerkamp, Alexander C. Sommerkamp, Petra Zeisberger, Paula Leonie Eiben, Andreas Narr, Aylin Korkmaz, Adriana Przybylla, Markus Sohn, Franciscus van der Hoeven, Kai Schönig, Andreas Trumpp

**Affiliations:** 1 Division of Stem Cells and Cancer, German Cancer Research Center (DKFZ) and DKFZ-ZMBH Alliance, Im Neuenheimer Feld 280, 69120 Heidelberg, Germany; 2 Heidelberg Institute for Stem Cell Technology and Experimental Medicine (HI-STEM gGmbH), 69120 Heidelberg, Germany; 3 Hopp Children’s Cancer Center Heidelberg (KiTZ), Heidelberg, Germany; 4 Pediatric Glioma Research Group, German Cancer Consortium (DKTK), German Cancer Research Center (DKFZ), Heidelberg, Germany; 5 Transgenic Service, German Cancer Research Center (DKFZ), Im Neuenheimer Feld 280, 69120 Heidelberg, Germany; 6 Central Institute for Mental Health, University of Heidelberg, 68159 Mannheim, Germany; 7 German Cancer Consortium (DKTK), 69120 Heidelberg, Germany

**Keywords:** Haematopoietic stem cells, Cell biology, Molecular biology, Stem cells

## Abstract

Poly(A) binding protein nuclear 1 (PABPN1) is known for its role in poly(A) tail addition and regulation of poly(A) tail length. In addition, it has been shown to be involved in alternative polyadenylation (APA). APA is a process regulating differential selection of polyadenylation sites, thereby influencing protein isoform expression and 3ʹ-UTR make-up. In this study, we generated an inducible *Pabpn1*^flox/flox^ mouse model using crRNA-tracrRNA:Cas9 complexes targeting upstream and downstream genomic regions, respectively, in combination with a long single-stranded DNA (ssDNA) template. We performed extensive in vitro testing of various guide RNAs (gRNAs) to optimize recombination efficiency for in vivo application. *Pabpn1*^flox/flox^ mice were generated and crossed to MxCre mice for validation experiments, allowing the induction of Cre expression in the bone marrow (BM) by poly(I:C) (pIC) injections. Validation experiments revealed successful deletion of *Pabpn1* and absence of PABPN1 protein. Functionally, knockout (KO) of *Pabpn1* led to a rapid and robust depletion of hematopoietic stem and progenitor cells (HSPCs) as well as myeloid cells, suggesting an essential role of *Pabpn1* in the hematopoietic lineage. Overall, the mouse model allows an inducible in-depth in vivo analysis of the role of PABPN1 and APA regulation in different tissues and disease settings.

## Introduction

Most mRNA molecules are co-transcriptionally polyadenylated in the nucleus. The transcribed mRNA strand is cleaved in an endonucleolytic process and the poly(A) tail is added to the 3ʹ-UTR^1^. Different multiprotein complexes are involved in this process. This polyadenylation step is a pre-requisite for nuclear export and the poly(A)-tail is regulating mRNA stability and translation^2,3^. One of the proteins involved in polyadenylation is poly(A) binding protein nuclear 1 (PABPN1). PABPN1 was initially identified as a protein important for poly(A) tail addition and regulation of poly(A) tail length^4–7^. Recently, it has also been shown to be involved in a process termed alternative polyadenylation (APA)^8–11^. The majority of genes harbor more than one polyadenylation site, and differential usage of these sites is termed APA. APA affects protein isoform expression, mRNA stability, translation efficiency and mRNA localization^1^. Several reports showed that PABPN1 is an important regulator of APA and extended these findings by associating mutated *Pabpn1* and deregulated APA to the myopathic disease OPMD^9,10^. Additional studies reported *Pabpn1* as an APA regulator in muscle cells, during muscle wasting, and in hematopoietic stem cells (HSCs)^8,11,12^. We showed previously that *Pabpn1* is important for mouse HSC function in the bone marrow (BM) and that deregulated APA patterns were observed upon *Pabpn1* knockdown (KD)^12^.

In the past, different transgenic mouse models were developed expressing mutated *Pabpn1,* which can be used to study the pathology of OPMD in mice^13,14^. While these mouse models allow analysis of the role of mutated PABPN1, their use to study the role of PABPN1 in APA in vivo remains limited. Several studies could show that not only mutation of *Pabpn1*, but also changes in PABPN1 protein levels lead to robust deregulation in APA^10,12^. In order to study PABPN1 and APA in vivo in different tissues and cell populations, an inducible PABPN1 knockout (KO) mouse model is needed. Vest et al. reported such a mouse model in 2017 and phenotyped heterozygous PABPN1 KO mice^14^. In this mouse model the integrated loxP sites flank exon 1 and 2, but not the *Pabpn1* promoter. However, several *Pabpn1* isoforms do not include exon 1 and 2 and thus this generated allele may not be considered a null. We therefore aimed to develop an inducible *Pabpn1* KO (null) mouse model that allows deletion of all expressed *Pabpn1* isoforms and introduced loxP sites flanking exon 3 and 4. The generation of transgenic mice was successful and the targeting strategy was verified by a proof-of-principle study demonstrating the deletion of *Pabpn1* in hematopoietic cells after expression of Cre under the control of the *Mx1* promoter.

## Results

### gRNA design and testing

**Figure 1:**
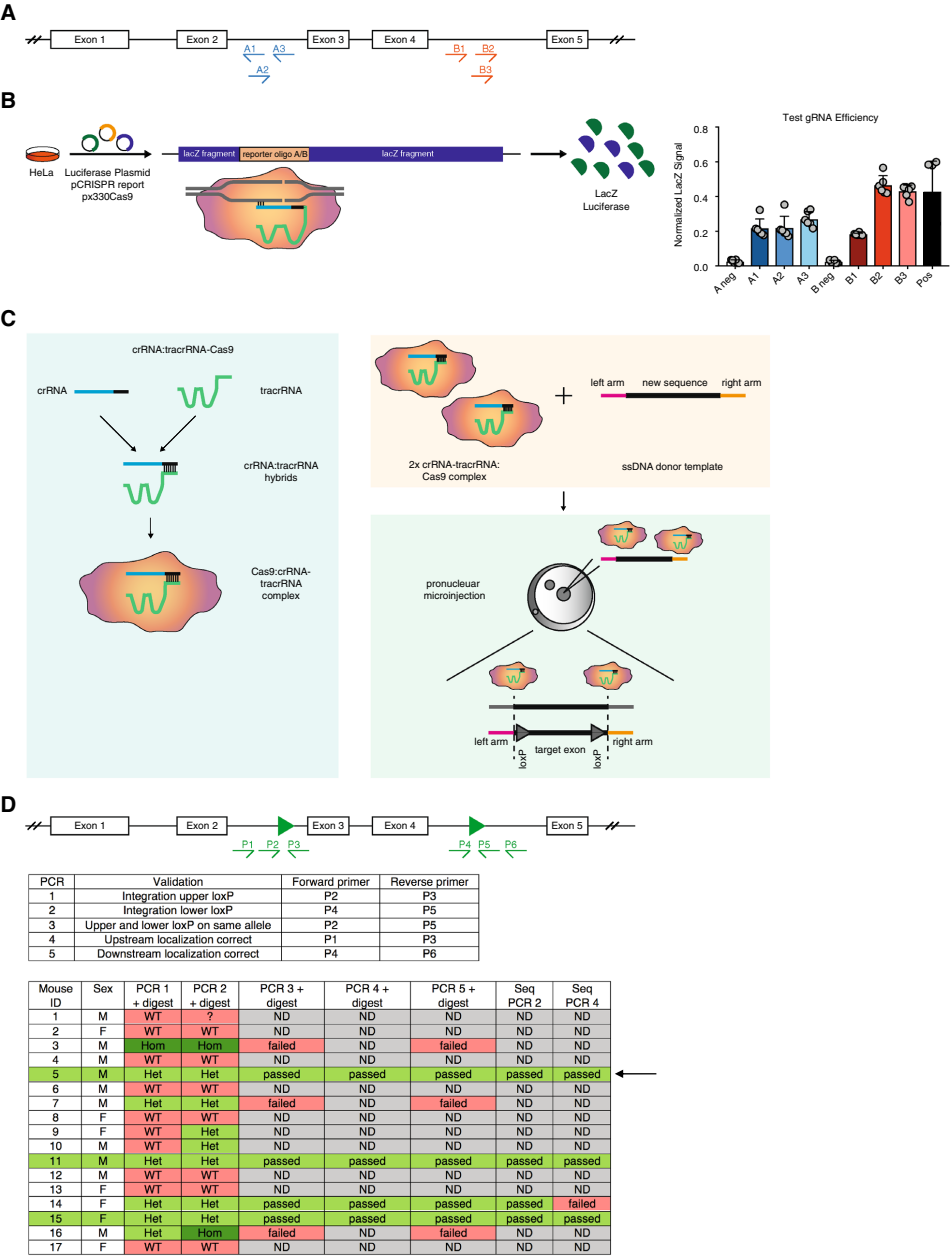
Generation of *Pabpn1*^flox/flox^ mice. (**A**) gRNA design based on *Pabpn1* isoform expression and PAM localization. (**B**) Schematic representation of the lacZ fragments in the pCRISPR report vector containing targeting sequence oligo A or B. Testing of gRNA efficiency. Normalized LacZ signal is shown. n = 6. n indicates number of biological replicates; 2 independent experiments were performed; mean + SD is shown. (**C**) Schematic representation of pronuclear injection of a long ssDNA donor template together with crRNA-tracrRNA:Cas9 ribonucleoprotein complexes into mouse zygotes. The crRNA-tracrRNA hybrids are pre-assembled and form a complex with Cas9 protein. These complexes are injected into the pronucleus of zygotes together with the ssDNA template. Two different crRNA-tracrRNA:Cas9 complexes are employed that cut upstream and downstream of the region of interest, respectively. The crRNA targets the complex to the respective target site by complementary base-pairing and Cas9 cleaves the DNA, generating a DNA DSB. The ssDNA strand functions as a donor template and is used for homology-directed repair (HDR), eventually resulting in the integration of loxP sites. (**D**) Schematic representation of the transgenic locus and localization of genotyping primers. Overview of *Pabpn1*^flox/flox^ mouse primers and genotyping results. *ND* not determined, *Het* heterozygous, *Hom* homozygous, *WT* wildtype. Drawings (**A–D**) were created using Adobe Illustrator 2022 (26.0.3).

The primary aim of this study was the generation and validation of a floxed *Pabpn1* transgenic mouse model that can be crossed to any Cre driver line to induce tissue-specific or inducible hetero- or homozygous KO of *Pabpn1*. To ensure loss of all protein-encoding isoforms, *Pabpn1* isoforms and functional domains were analyzed using Ensembl (www.ensembl.org). Several isoforms are reported, and all of them share expression of exon 3 and 4 of the *Pabpn1* isoform ENSMUST00000022808.13. In addition, the RNA binding domains are encoded in these exons. Therefore, we aimed to introduce loxP sites flanking exon 3 and 4 (Fig. 1A). For generation of transgenic mice, a CRISPR/Cas9-based approach was used^15,16^. 3 gRNAs per targeting locus were designed (upstream of exon 3: gRNAs A1–3; downstream of exon 4: gRNAs B1–3) using CRISPOR (http://crispor.tefor.net/)^17^ (Fig. 1A). The targeting sequences were located at least 250 bp up- or down-stream of the respective exon to exclude interference of the introduced loxP sites with splicing. To ensure efficient recombination in vivo, we performed pre-testing of gRNA efficiency in vitro.

Towards this end, we made use of a disrupted *lacZ* reporter gene harboring the respective targeting sequence (gRNAs A1–3 or gRNAs B1–3; Fig. 1B). Targeting sequences correspond to the *Pabpn1* introns targeted by the respective gRNAs and were inserted between the *lacZ* fragments in the pCRISPR report vector. gRNA oligonucleotides were annealed and cloned into the px330 Cas9 vector, enabling gRNA/Cas9 expression. To test gRNA efficiency, HeLa cells were transfected with the px330 Cas9 vector encoding for gRNA A1–3 or B1–3. Co-transfection with the respective pCRISPR reporter plasmid and a luciferase-encoding control plasmid (pUHC131.1) was performed. 24 h later, cells were lysed and LacZ as well as luciferase activity were measured. The normalized LacZ signal of the respective tested gRNA is a direct indicator of gRNA targeting efficiency. Testing revealed the highest efficiency for gRNAs A3 and B2 (Fig. 1B).

### Generation and genotyping of *Pabpn1*^flox/flox^ mice

We then made use of the Easi-CRISPR method described by Miura et al. and Quadros et al.^15,16^. Briefly, a long ssDNA donor template is injected together with pre-assembled CRISPR RNA—trans-activating RNA—CRISPR-associated protein 9 (crRNA-tracrRNA:Cas9) ribonucleoprotein complexes into mouse zygotes (Fig. 1C). The crRNA molecule directs the complex to the target site by complementary base-pairing, while the tracrRNA part is bound by Cas9. After binding to the target site, Cas9 cleaves the DNA, generating a DNA double-strand break (DSB). By providing template DNA strands, in this case a ssDNA donor template, the Cas9-induced DSB can be used for homology-directed repair (HDR). Excision and recombination take place in the 1-cell or early 2-cell state. crRNAs corresponding to gRNA sequences A3 and B2 and a long ssDNA megamer were ordered from IDT. In the 1126 nucleotide (nt) long megamer, a 60 nt homology arm was followed by the upper loxP site, exon 3 and 4 including intronic sequences, the lower loxP site and a 60 nt downstream homology arm. crRNA-tracrRNA:Cas9 complexes and the ssDNA template were injected into one of the pronuclei of C57Bl6/J zygotes, which were subsequently transplanted into pseudo-pregnant foster mice.

From two rounds of injections, 17 mice were born (Fig. 1D). In order to identify potential founder mice, we developed a stringent genotyping strategy. In a first step, integration of the upstream (us) and the downstream (ds) loxP site was evaluated by PCR1 and PCR2, respectively. Successful integration of loxP sites leads to a band shift of 36 bp. This size difference can be difficult to detect in gel analysis. Thus, in addition to loxP sites, we also introduced restriction enzyme sites during design of the ssDNA template. Only PCR products generated by amplification of the *Pabpn1* floxed sequence can be digested. Analysis revealed 7 animals with integration of both loxP sites (Fig. 1D, Supplementary Fig. 1A–D). Next, PCR3 and subsequent digestion was performed to ensure that both loxP sites were localized on the same allele (Fig. 1D, Supplementary Fig. 1E,F). To check for targeted integration in contrast to random integration, PCR4 (upper loxP) and PCR5 (lower loxP) and subsequent digestions were performed (Fig. 1D, Supplementary Fig. 1G–J). In total 4/17 mice passed the initial genotyping PCRs. To exclude the presence of point mutations, we performed amplicon sequencing of PCR2 and 4. In total, 3 animals (IDs: 5, 11, 15) passed this final QC (Fig. 1D). The mouse with ID5 was crossed to C57Bl6/J wildtype animals to generate heterozygous offspring. These mice were again extensively genotyped, and loxP integration was verified (data not shown). Heterozygous mice were crossed, generating homozygous *Pabpn1*^flox/flox^ mice.

### Generation of MxCre *Pabpn1*^flox/flox^ mice and validation of *Pabpn1* KO

**Figure 2:**
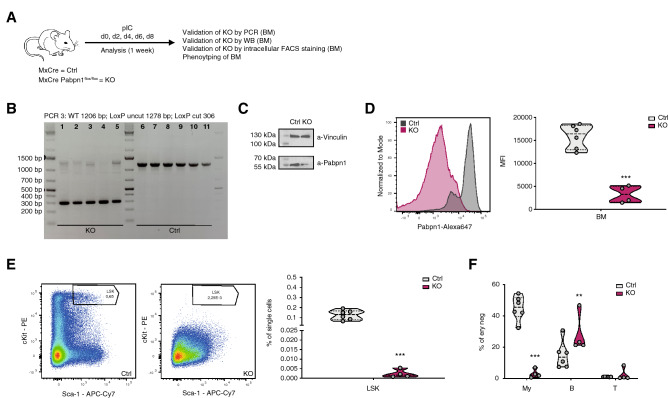
Generation of MxCre *Pabpn1*^flox/flox^ mice and validation of *Pabpn1* KO. (**A**) Workflow of MxCre *Pabpn1*^flox/flox^ treatment and analysis approach. (**B**) Gel images showing analysis of DNA recombination in MxCre *Pabpn1*^flox/flox^ and MxCre control mice. (**C**) KO validation by western blot analysis using ery-lysed total BM cells. Images were cropped, see Supplementary Fig. 2A for blots. (**D**) KO validation by intracellular flow cytometry analysis using ery-lysed total BM cells. (**E**) Flow cytometry-based analysis of the HSPC containing LSK population in the BM. (**F**) Flow cytometry-based analysis of hematopoietic lineages in the BM. For all experiments: Representative flow cytometry plots are shown. n = 4–6. n indicates number of biological replicates; 1 independent experiment; mean + SD is shown; unpaired Student’s t test (**D,E**); Two-way ANOVA (**F**); *p < 0.05; **p < 0.01; ***p < 0.001. LSK (Lin− Sca-1+ cKit+), T cells (T) (CD71− Ter119− GR1− CD11b− CD4/8+ B220−), B cells (B) (CD71− Ter119− GR1− CD11b− CD4/8− B220+), myeloid cells (My) (CD71− Ter119− GR1+ CD11b+). Drawings (**A**) were created using Adobe Illustrator 2022 (26.0.3).

To validate the function of the inducible *Pabpn1* KO mouse model, *Pabpn1*^flox/flox^ mice were crossed to the MxCre transgenic line (Ctrl) and backcrossed to generate MxCre *Pabpn1*^flox/flox^ mice (KO) (Fig. 2A). In MxCre mice, Cre expression is controlled by the interferon-response promoter *Mx1*^18^. Injections with the dsRNA analog poly(I:C) (pIC) can be used to activate Cre expression under the *Mx1* promoter, leading to efficient deletion of the floxed allele mainly in liver, spleen and hematopoietic cells^18^. Ctrl and KO mice were injected 5 times with pIC to induce Cre expression in the hematopoietic compartment. BM cells were isolated and used for KO validation and phenotypic characterization. PCR analysis of erythrocyte-lysed whole BM cells revealed highly efficient recombination, as expected (Fig. 2B). Strong reduction of PABPN1 protein in BM cells was confirmed by Western blot analysis and intracellular PABPN1 staining paired with subsequent flow cytometry analysis (Fig. 2C,D). Phenotypic analysis revealed depletion of the hematopoietic stem and progenitor cell (HSPC) containing Lineage− Sca-1+ c-Kit+ (LSK) compartment in the BM of *Pabpn1* KO mice (Fig. 2E). In addition, reduced frequencies of myeloid cells in the BM were observed, leading to a relative increase in B cell frequencies (Fig. 2F). Taken together, we successfully validate the function of our *Pabpn1*^flox/flox^ mouse model.

### Characterization of MxCre *Pabpn1*^flox/flox^ mice

**Figure 3:**
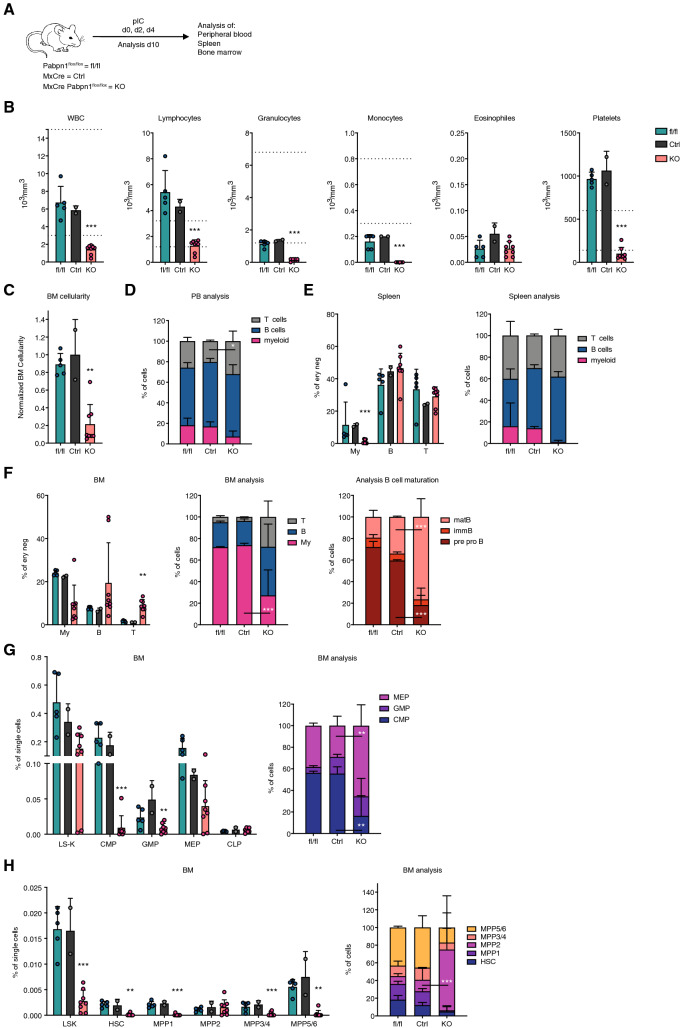
Characterization of MxCre *Pabpn1*^flox/flox^ mice. (**A**) Workflow of MxCre *Pabpn1*^flox/flox^ treatment and analysis approach. (**B**) Hemavet analysis of peripheral blood. (**C**) Normalized non-ery lysed BM cellularity. (**C–E**) Flow cytometry-based analysis of hematopoietic lineages in the blood (**C**), spleen (**D**) and BM (**F**). (**G**) Flow cytometry-based analysis of the progenitor containing LS-K population and CLP in the BM. (**H**) Flow cytometry-based analysis of the HSPC containing LSK population in the BM. For all experiments: n = 2–8. n indicates number of biological replicates; 1–3 independent experiments; mean + SD is shown; unpaired Student’s t test (% of ery neg or % of single cells panels) or Two-way ANOVA (% of cells panels); *p < 0.05; **p < 0.01; ***p < 0.001; for statistical analysis KO was compared to Ctrl. T cells (T) (CD71− Ter119− GR1− CD11b− CD4/8+ B220−), B cells (B) (CD71− Ter119− GR1− CD11b− CD4/8− B220+), myeloid cells (My) (CD71− Ter119− GR1+ CD11b+), mature B cells (matB) (CD71− Ter119− GR1− CD11b− CD4/8− B220^high^ IgM+), immature B cells (immB) (CD71− Ter119− GR1− CD11b− CD4/8− B220^low^ IgM+), pre pro B cells (pre pro B) (CD71− Ter119− GR1− CD11b− CD4/8− B220^low^ IgM−), LS-K (Lin− Sca-1− cKit+), CMP (LS-K IL7R− CD34+ CD16/32−), GMP (LS-K IL7R− CD34+ CD16/32+), MEP (LS-K IL7R− CD34− CD16/32−), CLP (Lin− Sca-1^low^ c-Kit^low^ IL7R+), LSK (Lin− Sca-1+ cKit+), HSC (LSK CD48− CD150+ CD34−), MPP1 (LSK CD48− CD150 + CD34+), MPP2 (LSK CD48+ CD150+), MPP3/4 (LSK CD48+ CD150−), MPP5/6 (LSK CD48− CD150−). Drawings (**A**) were created using Adobe Illustrator 2022 (26.0.3).

In order to better and more extensively characterize the effects of homozygous *Pabpn1* KO in the hematopoietic system, we used a milder pIC treatment protocol, with 3 instead of 5 injections, and performed analysis already at day 10 (Fig. 3A). This treatment regime did not lead to complete loss of LSK and LS-K cells as observed with the previous treatment approach (Fig. 2) and thus allowed us to perform a more in-depth analysis of the hematopoietic phenotype. MxCre (Ctrl), MxCre *Pabpn1*^flox/flox^ mice (KO) and *Pabpn1*^flox/flox^ mice, not carrying the MxCre allele (fl/fl) and serving as an additional control, were injected with pIC and analyzed. Hemavet analysis revealed reduced numbers of mature blood cells, with the strongest effect observed in the myeloid lineage and platelet counts in KO animals (Fig. 3B, Supplementary Fig. 3A). This reduction in mature blood cells was accompanied by a strongly reduced total BM cellularity (Fig. 3C). Further analysis of the blood, spleen and BM confirmed significantly reduced numbers of myeloid cells (Fig. 3D–F). Interestingly, we also observed reduced numbers of pre-pro B cells and immature B cells, while numbers of mature B cells were not affected (Fig. 3F). Analysis of T cell maturation in the thymus revealed a decrease in the immature double negative (DN) population (Supplementary Fig. 3B). In depth characterization of BM of *Pabpn1* KO mice revealed reduced frequency of more committed progenitors contained in the Lin− Sca-1− c-Kit+ (LS-K) compartment (Fig. 3G). We observed reduced numbers of common myeloid progenitors (CMP) and granulocyte–macrophage progenitors (GMP), while megakaryocyte-erythrocyte progenitors (MEP) and common lymphoid progenitors (CLP) were unchanged. In addition, analysis of erythroid maturation revealed an increase in the more mature erythroblast populations and a decrease in maturing erythroblasts (Supplementary Fig. 3C). The HSPC containing LSK compartment was massively reduced in KO animals, all HSC and MPP populations except MPP2 were depleted (Fig. 3H). Overall, we observed strong effects on the hematopoietic system following depletion of *Pabpn1*. Especially mature myeloid cells, myeloid progenitors and HSPCs were particularly dependent on *Pabpn1* at the analyzed timepoint.

## Discussion

Using the Easi-CRISPR technique^15,16^, we successfully generated an inducible *Pabpn1* KO mouse model, with loxP sites flanking exon 3 and 4. We achieved a targeting efficiency of 18% (3/17 animals). In our hands, the Easi-CRIPSR strategy was a quick and reliable approach to generate floxed transgenic animals.

A common issue with CRISPR-Cas9 targeting approaches are off-target effects^19^. gRNAs were chosen based on their reported off-target score. gRNA A3 was predicted to have 90 potential off-targets with at least 3 mismatches and gRNA B2 presented 100 anticipated off-targets with at least 2 mismatches. A high number of mismatches decreases the likelihood of off-target mutations. Because of the absence of homologous regions, an off-target event would lead to a mutation, but not to loxP site integration. All of the predicted off-targets are localized on other chromosomes than *Pabpn1* leading to loss of mutation by further crossing of the generated mice. We therefore did not systematically screen for off-target events. Overall, we minimized the likelihood of off-target events by gRNA choice and backcrossing of mice.

In contrast to the transgenic inducible *Pabpn1* KO mouse model developed by Vest et al., loxP sites in our mouse model flank exon 3 and 4, ensuring deletion of all encoded *Pabpn1* isoforms^14^. To date it remains unclear which of the reported *Pabpn1* isoforms are translated in addition to full length *Pabpn1* (ENSMUST00000022808.14). Thus, the model reported by Vest et al. might represent a bona fide null KO mouse model. The targeting strategy of our *Pabpn1* flox mouse model presented here, however, ensures depletion of all the potential isoforms.

We functionally validated our mouse model by crossing *Pabpn1* flox mice to the MxCre line followed by Cre induction using pIC, leading to efficient deletion of the floxed allele in hematopoietic cells. Recombination was observed on the DNA level, and reduced levels of PABPN1 protein in total BM cells was confirmed by Western blot and intracellular flow cytometry analysis. Unfortunately, analysis of sorted cell populations was hindered by the strong phenotype observed in hematopoietic cells leading to a loss of the respective cell populations. The residual level of PABPN1 protein still detected in total KO BM cells is presumably due to the presence of non-hematopoietic cell types not expressing Cre.

As a proof-of-concept experiment to characterize *Pabpn1* KO mice, we performed extensive profiling of BM cells. This revealed depletion of HSPCs and reduced frequencies of myeloid progenitors and mature myeloid cells in the blood, thymus, BM and spleen. In addition, B and T cell maturation was impaired and reduced numbers of non-mature B and T cells were observed. Overall, short-lived mature blood cells and progenitors seem to be more strongly affected by loss of *Pabpn1*. Interestingly, 10 days after the first pIC injection also the HSPC compartment was massively depleted. This compartment usually contains highly quiescent long-lived cell subsets^20^. The HSPC compartment could directly be affected by loss of *Pabpn1* or loss of mature cells could lead to activation of this compartment as observed for other genes^21^. This activation in the absence of *Pabpn1* could cause cell death of HSPCs. Overall, our findings are in line with our previous HSPC *Pabpn1* KD studies, in which we observed impaired HSC function^12^. In the future, additional studies need to be conducted to better understand the kinetics and the mechanism of the observed phenotype.

Overall, we describe the successful generation of an inducible Pabpn1 KO mouse model. In the future, this mouse model can be used to facilitate functional in vivo research on the APA regulator PABPN1 using different tissue-specific Cre drivers and disease models.

## Methods

### Mice

C57BL/6J, MxCre, *Pabpn1*^flox/flox^ and MxCre *Pabpn1*^flox/flox^ mice were bred in-house in the animal facility of the DKFZ under specific-pathogen-free (SPF) conditions in individually ventilated cages (IVCs). All animal procedures were performed according to protocols approved by the German authorities (official licensing committee), *Regierungspräsidium Karlsruhe* (Nr. A-23/17, Z110/02, DKFZ299, G-256/16, G-24/19, G-250/20) and were carried out in compliance with the ARRIVE guidelines. 6–20 weeks old male and female animals were used. No blinding or randomization of animals during experiments was performed.

For testing of the generated mouse model, all animals were injected intraperitoneally with pIC (100 μg pIC in PBS). Figure 2: Injections at d0, d2, d4, d6, d8; analysis after 1 week. Figure 3: Injections at d0, d2, d4; analysis at d10.

### Testing of gRNA efficiency

#### Cloning of the px330 Cas9 vector

Oligos encoding the target-specific part of the crRNA were ordered for expression of full-length gRNAs (Supplementary Table 1, Sigma-Aldrich; design gRNA A: http://crispor.tefor.net/crispor.py?batchId=aQ9aKiq6R1spZW5ypsBG; design gRNA B: http://crispor.tefor.net/crispor.py?batchId=idFOo1VhuwIGjPTeLMe3) in the px330 Cas9 vector (42230, Addgene). The respective oligos were annealed for 2 h (starting at 95 °C continuously cooled to RT). The px330 Cas9 vector was digested with BbsI enzyme for 2 h at 37 °C. Annealed oligos and the digested px330 Cas9 vector were purified by agarose gel electrophoresis (2% for oligos; 1% for vector). Gel extraction using the QIAEX II Gel Extraction Kit (Qiagen) according to manufacturer’s guidelines (elution in 20 µl) was performed. 100 ng digested vector was ligated with 1.2 ng annealed oligos for 1.5 h at 22 °C using T4 ligase (New England BioLabs Inc.). Bacterial (*E. coli*) transformation was performed with the ligated px330 Cas9 vectors. Transformed bacteria were plated on agar plates (containing 100 µg/ml ampicillin) and incubated overnight at 37 °C. Colonies were picked and colony PCR (Supplementary Table 2) was performed (Supplementary Table 3). Successful integration of target sequence oligos in the px330 Cas9 vector leads to a product of approximately 500 bp. Presence of this product was verified by agarose gel electrophoresis (1%). Positive clones were used for overnight culture and subsequent DNA plasmid Mini-prep using the NucleoSpin Plasmid Kit (Macherey Nagel) according to manufacturer’s guidelines was performed. Enzymatic digestion was used to test for oligo integration using the enzymes BbsI and EcoRI (both New England BioLabs Inc.). Re-ligation of px330 Cas9 vector without oligo integration leads to 2 products after the digestion step (3.2 kb and 5.3 kb). Successful oligo integration leads to abrogation of the BbsI restriction enzyme sites and results in the generation of only one 8.5 kb product. Digestion was performed for 2 h at 37 °C. Product size was analyzed by agarose gel electrophoresis (1%).

#### Cloning of the pCRISPR-report vector

In addition, oligos encoding for target sequences (Supplementary Table 1, Sigma-Aldrich) were ordered for cloning into the pCRISPR-Report vector (pTAL-Rep)^17^ for gRNA testing. Oligos were annealed and purified by gel extraction as previously described (see “Cloning of the px330 Cas9 vector” section). The pCRISPR-Report vector was digested using BstBI and NruI and purified by gel extraction as previously described (see “Cloning of the px330 Cas9 vector” section). 100 ng digested vector was ligated with 1.2 ng annealed oligos for 1.5 h at 22 °C using T4 ligase (New England BioLabs). Bacterial (*E. coli*) transformation was performed using the ligated pCRISPR-Report vectors encoding for lacZ fragments separated by targeting sequences that are equivalent to the mouse *Pabpn1* targeting sequences for upstream gRNAs A and downstream gRNAs B. Transformed bacteria were plated on agar plates (containing 100 µg/ml ampicillin) and incubated overnight at 37 °C. Colonies were picked and colony PCR was performed (Supplementary Tables 3, 4). Successful integration of oligos in the pCRISPR-Report vector leads to a product of approximately 900 bp length and was verified by agarose gel electrophoresis (1%). Positive clones were used for overnight cultures and subsequent DNA plasmid Mini-prep using the NucleoSpin Plasmid Kit (Macherey Nagel) according to manufacturer’s protocols was performed. Enzymatic digestion was used to test for oligo integration using the enzymes SnaBI (pCRISPR-Report for testing of A gRNAs) or EcoRI (pCRISPR-Report for testing of B gRNAs) (all New England BioLabs). Re-ligation of pCRISPR-Report vector without oligo integration leads to 1 product after the digestion step (7.4 kb). Successful oligo integration leads to the presence of the respective restriction enzyme sites and the generation of two products (A: 0.8 kb + 6.6 kb; B: 1.2 kb + 6.2 kb). Digestion was performed for 2 h at 37 °C. Product size was analyzed by agarose gel electrophoresis (1%). Positive clones were selected and overnight culture and subsequent DNA plasmid Midi-prep using the Macherey Nagel NucleoBond Xtra Midi Plasmid Kit according to manufacturer’s protocols was performed.

#### Reporter assay

25,000 HeLa cells/well were seeded in 12-well plates in DMEM GlutaMAX (+ 10% FCS, + 1% Pen/Strep). Cells were incubated for 2–3 h. Transfection mix was set up (Supplementary Table 5) and mixed with 100 µl RotiFect mix (5 µl RotiFect (Carl Roth) in 100 µl Opti-MEM I) and incubated for 30 min at RT. 200 µl transfection mix were added per well, and cells were incubated overnight at 37 °C. Cells were washed with PBS and lysed using 1× passive lysis buffer for 3 min at RT. Lysates were centrifuged at 13,000 rpm for 5 min at 4 °C. 30 µl supernatant per replicate and condition were transferred to a fresh Eppendorf tube, and 700 µl Z-buffer (1.60 g Na2HPO4.7H2O, 0.55 g NaH2PO4.H2O, 0.075 g KCl, 0.012 g MgSO4, pH 7.0, in 100 ml dH2O, 0.35% 2-Mercaptoethanol) were added and mixed. Subsequently, 200 µl ONPG solution (0.55 g ONPG in 100 ml dH2O) were added per tube and incubated at RT until a yellow color-shift was observed. Reaction was stopped using 500 µl 1 M NaOH per tube, and the signal was measured (420 nm). For luciferase measurements, 10 µl of lysate per replicate and condition were pipetted into a white 96-well plate. This plate was measured in a luciferase detection instrument (Perkin Elmer Wallac 1420 Victor2 Microplate Reader). To calculate normalized signals, the following equation was used: LacZ/Luciferase*mean(Luciferase).

### Easi-CRISPR

crRNA, tracrRNA and ssDNA template molecules were ordered from IDT (Supplementary Table 6). crRNA and tracrRNA were reconstituted in microinjection buffer (10 mM TrisHCl 1 mM EDTA pH 7.5 in dH2O) to a final concentration of 100 µM. Cas9 protein was diluted in microinjection buffer to a final concentration of 500 ng/µl. The ssDNA template was reconstituted in 30 µl H_2_O. All stocks were aliquoted and stored at − 80 °C. crRNA and tracrRNA were diluted to 6.1 µM, and 15 µl crRNA + 15 µl tracrRNA were mixed and incubated for 2 min at 94 °C and 10 min at RT. The generated 3.1 µM crRNA:tracrRNA mixes were aliquoted and stored at − 80 °C. For pronucleus injections, 1 µl crRNA_A3:tracrRNA (final 0.31 µM) was mixed with 1 µl crRNA_B2:tracrRNA (final 0.31 µM), 1 µl ssDNA template (final 10 ng/µl), 1 µl Cas9 protein (final 50 ng/µl) and 6 µl microinjection buffer. Injections were performed by the Transgenic Service of the DKFZ.

### Genotyping

For genotyping of offspring, tails were digested, and tail DNA was purified using the Qiagen DNeasy Blood and Tissue Kit following manufacturer’s instructions (elution in 100 µl). 5 different PCR and digestion approaches were used to validate loxP integration (Supplementary Table 7). DreamTaq Green PCR Mastermix (Thermo Fisher Scientific) was used for PCR amplification (Supplementary Tables 8–10). PCR products were analyzed by agarose gel electrophoresis (Supplementary Table 11) and purified using the QIAquick PCR Purification Kit (Qiagen) according to manufacturer’s guidelines. Enzyme digestion was performed at 37 °C for 1.5 h and analyzed using agarose gel electrophoresis (Supplementary Table 12). For mice exhibiting correct integration, PCR 2 and PCR 4 were repeated, and agarose gel electrophoresis was performed. The loxP band was excised and purified using the Qiagen QIAquick Gel Extraction Kit according to manufacturer’s guidelines. Purified PCR products were sent for sequencing by Eurofins together with the respective rev primer (PCR 2) and fwd primer (PCR 4) to exclude presence of point mutations.

### Cell suspension and flow cytometry

Mouse BM was isolated from pooled femora, tibiae and ilia by gentle crushing in PBS using a mortar and pistil. Lysis of erythrocytes was performed using ACK Lysing Buffer (Thermo Fisher Scientific). 1 × 10^6^ cells were snap-frozen and stored at − 80 °C as a cell pellet for DNA or protein extraction. Non-ery lysed blood was used for Hemavet analysis. Ery-lysed peripheral blood, spleen and BM cells were used for flow cytometry stainings and cell suspensions were stained for 30 min.

For flow cytometry analysis of HSPCs and differentiated cells (Fig. 2) the following monoclonal antibodies were used: HSPC staining—anti-lineage [anti-CD4 (clone GK1.5), anti-CD8a (53–6.7), anti-CD11b (M1/70), anti-B220 (RA3-6B2), anti-GR1 (RB6-8C5) and anti-TER119 (Ter-119)] all PB; anti-CD117/c-Kit (2B8)-PE; anti-Ly6a/Sca-1 (D7)-APC-Cy7; staining differentiated cells—anti-CD4 (GK1.5)-PE-Cy7, anti-CD8a (53–6.7)-PE-Cy7, anti-CD11b (M1/70)-APC-Cy7, anti-B220 (RA3-6B2)-AF700, anti-GR1 (RB6-8C5)-APC, anti-Ter119 (Ter-119)-PB; anti-CD71 (R17217)-PE.

For flow cytometry analysis of Blood, Spleen and BM (Fig. 3) the following monoclonal antibodies were used: HSPC staining—anti-lineage [anti-CD4 (clone GK1.5), anti-CD8a (53–6.7), anti-CD11b (M1/70), anti-B220 (RA3-6B2), anti-GR1 (RB6-8C5) and anti-TER119 (Ter− 119)] all PE-Cy7; anti-CD117/c-Kit (2B8)-BV711; anti-Ly6a/Sca-1 (D7)-APC-Cy7; anti-CD34 (RAM34)-FITC; anti-CD150 (TC15-12F12.2)-PE-Cy5; anti-CD48 (HM48-1)-PB; anti-CD16/32 (93)-APC; anti-CD127 (A7R34)-PE; staining differentiated cells—anti-CD4 (GK1.5)-PE-Cy7, anti-CD8a (53–6.7)-PE-Cy7, anti-CD11b (M1/70)-APC-Cy7, anti-B220 (RA3-6B2)-AF700, anti-GR1 (RB6-8C5)-APC, anti-Ter119 (Ter-119)-PB; anti-CD71 (R17217)-PE; anti-IgM (II/41)-PE-Cy5.

Monoclonal antibody conjugates were purchased from eBioscience or BioLegend.

For analysis of KO efficiency, BM cells were fixed with BD Cytofix/Cytoperm Buffer (Beckton Dickinson). Subsequently, intracellular PABPN1 (AF647 anti-Pabpn1 Clone EP3000Y, abcam) staining was performed using PermWash solution (Beckton Dickinson).

### Analysis of in vivo recombination

For analysis of in vivo recombination, BM cell pellets were used. DNA was purified using the Qiagen DNeasy Blood and Tissue Kit following manufacturer’s instructions (elution in 75 µl). PCR 3 was used for analysis of DNA recombination (Supplementary Table 7) using DreamTaq Green PCR Mastermix for PCR amplification (Supplementary Tables 8, 10).

### Western blot

For Western Blot analysis equal number of cells from Ctrl or KO mice were taken and resuspended in RIPA lysis buffer containing 5 mM EDTA (ThermoFisher), 1× Halt Protease & Phosphatase Inhibitor Cocktail (ThermoFisher), and 1 mM PMSF (ThermoFisher). Lysates were vortexed, incubated on ice for 30 min and then centrifuged at 15,000×*g* at 4 °C for 15 min. Supernatant was transferred to a fresh tube and lysates were stored at − 20 °C. Protein concentration of the samples were assessed using the Pierce BCA Protein Assay Kit (ThermoFisher). NuPAGE LDS sample buffer (ThermoFisher) and NuPAGE Sample Reducing Agent (ThermoFisher) were added to 20 µg protein and heated at 70 °C for 10 min. Samples were separated on Criterion TGX Stain-Free Precast Gels (BioRad) and transferred to PVDF membranes (BioRad). Western Blotting was performed according to the BioRad protocol. Blots were stripped using Restore Western Blot Stripping Buffer (ThermoFisher). The following primary and secondary antibodies were used for western blot analyses: Pabpn1 (JM11–28; ThermoFisher), Vinculin (Cell Signaling 4650), HRP-linked anti-rabbit IgG (Cell Signaling 7074). Blots were developed with the Clarity or Clarity Max ECL Western Blotting Substrates (BioRad).

### Statistical analysis

Statistical analysis was performed by unpaired Student’s t test or two-way ANOVA without correction for multiple comparison (Fisher LSD test). Significance levels were set at *p < 0.05, **p < 0.01 and ***p < 0.001. GraphPad Prism was used for statistical analysis.

## Supplementary Information

Supplementary Information.
