# Supplementary material for: CRISPR-Cas9 mediated generation of a conditional poly(A) binding protein nuclear 1 (Pabpn1) mouse model reveals an essential role for hematopoietic stem cells
Source: Sci Rep. 2022 May 3;12:7181. doi: 10.1038/s41598-022-11203-x (PMC9065150; doi:10.1038/s41598-022-11203-x)
Supplement: Supplementary file 1 — Supplementary Information. [file 41598_2022_11203_MOESM1_ESM.pdf]

CRISPR-Cas9 mediated generation of a conditional Poly(A) binding protein nuclear 1 (*Pabpn1*) mouse model reveals an essential role for hematopoietic stem cells

Pia Sommerkamp[1,2], Alexander Sommerkamp[3,4], Petra Zeisberger[1,2], Paula Leonie Eiben[1,2], Andreas Narr[1,2], Aylin Korkmaz[1,2], Adriana Przybylla[1,2], Markus Sohn[1,2], Franciscus van der Hoeven[5], Kai Schönig[6], Andreas Trumpp[1,2,7,*]

[1] Division of Stem Cells and Cancer, German Cancer Research Center (DKFZ) and DKFZ-ZMBH Alliance, 69120 Heidelberg, Germany

[2] Heidelberg Institute for Stem Cell Technology and Experimental Medicine (HI-STEM gGmbH), 69120 Heidelberg, Germany

[3] Hopp Children's Cancer Center Heidelberg (KiTZ), Heidelberg, Germany

[4] Pediatric Glioma Research Group, German Cancer Consortium (DKTK), German Cancer Research Center (DKFZ), Heidelberg, Germany

[5] Transgenic Service, German Cancer Research Center (DKFZ), Im Neuenheimer Feld 280, 69120 Heidelberg, Germany

[6] Central Institute for Mental Health, University of Heidelberg, 68159, Mannheim, Germany

[7] German Cancer Consortium (DKTK), 69120 Heidelberg, Germany

Corresponding Author:

Prof. Dr. Andreas Trumpp
Im Neuenheimer Feld 280
D-69120 Heidelberg
Tel: +49 6221 42 3901
Fax: +49 6221 42-3902
E-Mail: a.trumpp@dkfz.de

Supplementary Table 1: gRNA and reporter oligos

Oligo	Forward sequence	Reverse sequence
gRNA A1	CACCgagctgataatcgccccaaga	AAACtcttggggcgattatcagctC
gRNA A2	CACCgttcttggggcgattatcagc	AAACgctgataatcgccccaagaaC
gRNA A3	CACCgttcctaagtcctaactaaa	AAACtttagttaggacttaggaac
gRNA B1	CACCgttaaactgggattagacttc	AAACgaagtctaatcccagtttaaC
gRNA B2	CACCgtgactggcctaacttgagct	AAACagctcaagttaggccagtcaC
gRNA B3	CACCggcctaacttgagctgggcg	AAACcgcccagctcaagttaggcc
Reporter A	cgGTCTGTACCCTTCTTGGGGC<br>GATTATCAGCTGGCATACgtaTG<br>GAGACCCTTTAGTTAGGACTTA<br>GGAACTGACTG	CAGTCAGTTCCTAAGTCCTAACTAA<br>AGGGTCTCCAtacGTATGCCAGCTG<br>ATAATCGCCCCAAGAAGGGTACAG<br>AC
Reporter B	cgCTCAGAATTAAACTGGGATTA<br>GACTTCAGGTTTATACCACCAT<br>GACTGGCCTAACTTGAGCTGGG<br>CGTGGTGGaattcg	cgaattCCACCACGCCCAGCTCAAGT<br>TAGGCCAGTCATGGTGGTATAAAC<br>CTGAAGTCTAATCCCAGTTTAATTC<br>TGAG

Supplementary Table 2: Colony PCR px330 Cas9 vector, Fisher Bioreagents *Taq* DNA Polymerase (Thermo Fisher Scientific), Buffer B and $MgCl_2$ provided with polymerase

Component	Amount (µl)
Buffer B	5
$MgCl_2$ (25 mM)	5
Fwd primer (respective fwd gRNA oligo)	0.3
Rev primer (px330_rev: gtactgccaagtaggaaagtcc)	0.3
dNTPs (10 mM)	0.5
TaqPol	0.5
H_2O	38.4

Supplementary Table 3: Colony PCR amplification program

Step	Temperature	Time	Number of cycles
Denaturation	95°C	5 min	
Amplification	95°C<br>62°C<br>72°C	20 s<br>30 s<br>30 s	30
Final elongation	72°C	2 min	
Storage	4°C	∞	

Supplementary Table 4: Colony PCR pCRISPR-Report vector, Fisher Bioreagents *Taq* DNA Polymerase (Thermo Fisher Scientific), Buffer B and $MgCl_2$ provided with polymerase

Component	Amount (µl)
Buffer B	5
$MgCl_2$ (25 mM)	5
Fwd primer (respective fwd reporter oligo)	0.3
Rev primer (lacZ_rev: ttacccgtaggtagtcacgca)	0.3
dNTPs (10 mM)	0.5
TaqPol	0.5
H_2O	38.4

Supplementary Table 5: Transfection mix

Component	Amount
pUHC131.1 (Luciferase Plasmid)	200 ng
px330 Cas9	400 ng
pCRISPR-Report	400 ng
Opti-MEM I	Fill up to 100 µl

Supplementary Table 6: IDT constructs

**Construct**	**Sequence**
crRNA A3	GTTCCTAAGTCCTAACTAAA
crRNA B2	TGACTGGCCTAACTTGAGCT
ssDNA megamer	GGCGATTATCAGCTGGCATTTGACCTTCAAGTCTTAACACTTTTCAGT TGGAGACCCTTTATAACTTCGTATAGCATACATTATACGAAGTTATaAG cTTAGGACTTAGGAACTGACTGGTGGCTGTAAGGGAGGGAGTTCTTT AAAAATGTTAGATTTCAGTCCATTCTGTTTTGTTTGCTTTGAGGCATTT CAGCTAAAGCTGAATAGAGACTCGACTTGAAAAACGTAAAATCTTAAC ACCGTTTTTTATGGTCTTTTAAGAGGTAGTTTCTCTAATGTATATTGAA ACCTACTATGTGCTAAACACTGTTATAGGTAATTGGTATAGTAGAAGT ATACCCAAAATTTAGCCTGCGGGTGGAAGTGTGTGTGTATTGATCGA GCAGAAGATGTGCTTGCCTAAGGTAATTTTTTTCCCCTCTAAAAGCTG GCCCAGTGATCATGTCTCTTGAGGAGAAGATGGAGGCTGATGCCCGC TCTATCTACGTTGGCAATGTATGTATCAAGGAATAGTCTGCTTTGGGT TTTGGGGGGGACTTTTGTTTCTTATTCCTTACTTTGTTATGTGTTCAAA TTGGAGTTTCCCCAGTACTTGTCAAGTGTTCCTTTCCTTAGGTGGACT ATGGTGCAACAGCAGAAGAGCTGGAAGCCCATTTTCATGGCTGTGGT TCAGTCAACCGTGTTACTATACTCTGTGACAAATTTAGTGGCCATCCC AAAGGGTAAGTAGGAGGATAAGTTGAGATCATTTTACTCACATTTTAA AAATACGTGAAAAATACATGAGCTCGGAATCGAACCTAGGACCTTGAA CATGCTGATGTGTGCTGTCAATCTAGTCAAAAGAGTGTTAACTCGTTT TAAAGGTTCTTATATATGTGGTCGGTTGGTTTTGTTTTCAAGTCAGAGT TTCTCTGTTTAACCCTGGCTGTACTGGAATTACCTCTATAGACCAGGC TGGCCTTAAACTCAGAATTAAACTGGGATTAGACTTCAGGTTTATACC ACCATGACTGGCtCTAgACTTGAATAACTTCGTATAGCATACATTATAC GAAGTTATGCTGGGCGTGGTGGTGCACGCCTTTAATCCCAGCACTTG GGAGACAGAGGCAGGGATTTC

Supplementary Table 7: Overview genotyping PCRs and digestion approaches (P: program, all enzymes from New England BioLabs)

PCR	Validation	Forward primer	Reverse primer	P	Enzyme
PCR 1	Integration upper loxP	CGATTATCAGCT GGCATTTGACC	GGCAAGCACATCT TCTGCTCG	P 1	HindIII-HF
PCR 2	Integration lower loxP	ACCTTGAACATG CTGATGTGTGC	GAGACACTTAACA GGCAAAACCTAC	P 1	XmnI
PCR 3	Upper and lower loxP on same allele	CGATTATCAGCT GGCATTTGACC	GAGACACTTAACA GGCAAAACCTAC	P 2	HindIII-HF + XmnI
PCR 4	Upstream localization correct	AAGCCGGGGAC CTTGAATG	GGCAAGCACATCT TCTGCTCG	P 2	HindIII-HF
PCR 5	Downstrea m localization correct	ACCTTGAACATG CTGATGTGTGC	ACTCTCACTGCCA CCAACTCC	P 2	XmnI

Supplementary Table 8: Genotyping PCR mastermix

Component	Amount
DreamTaq Green PCR Mastermix	25 µl
Fwd primer (100 µM)	0.3 µl
Rev primer (100 µM)	0.3 µl
DNA	100 ng
H_2O	up to 50 µl

Supplementary Table 9: PCR program 1 (P 1)

Step	Temperature	Time	Number of cycles
Denaturation	95°C	2 min	
Amplification	95°C<br>62°C<br>72°C	30 s<br>30 s<br>45 s	35
Final elongation	72°C	5 min	
Storage	4°C	∞	

Supplementary Table 10: PCR program 2 (P 2)

Step	Temperature	Time	Number of cycles
Denaturation	95°C	2 min	
Amplification	95°C<br>62°C<br>72°C	30 s<br>30 s<br>1 min	35
Final elongation	72°C	5 min	
Storage	4°C	∞	

Supplementary Table 11: Expected amplicon sizes

PCR	Wildtype (bp)	loxP (bp)
PCR 1	365	401
PCR 2	435	471
PCR 3	1206	1278
PCR 4	562	598
PCR 5	1477	1513

Supplementary Table 12: Expected product sizes after digestion

PCR	Wildtype (bp)	loxP (bp)
PCR 1	365	95 + 306
PCR 2	435	228 + 242
PCR 3	1206	Both alleles: 93 + 940 + 244<br>Upstream only: 93 + 1184<br>Downstream only: 1033 + 244
PCR 4	562	254 + 308
PCR 5	1477	231 + 1282

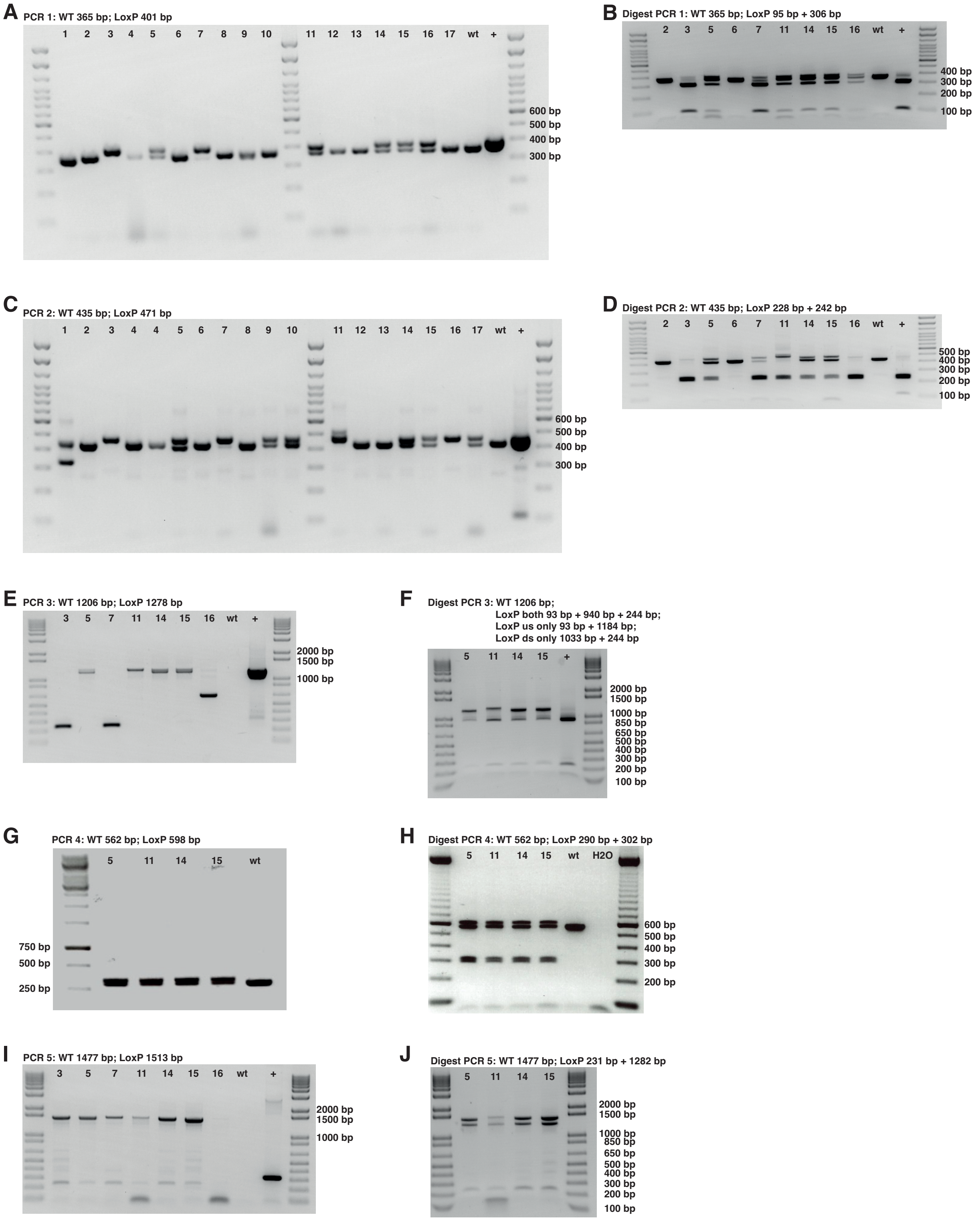

Supplementary Figure 1: Generation of Pabpn1$^{flox/flox}$ mice. Gel images showing genotyping results for PCR 1-5 (A, C, E, G, I) and digest of PCR 1-5 (B,D,F,H,J).

A

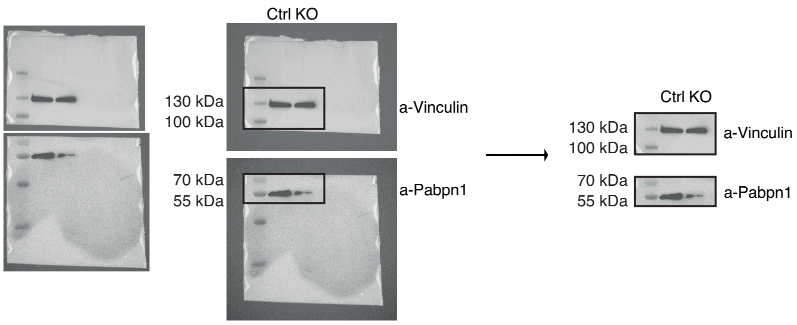

Supplementary Figure 2: (A) Generation of Figure 2C. Membrane was cut in two pieces after transfer and before addition of primary antibodies.

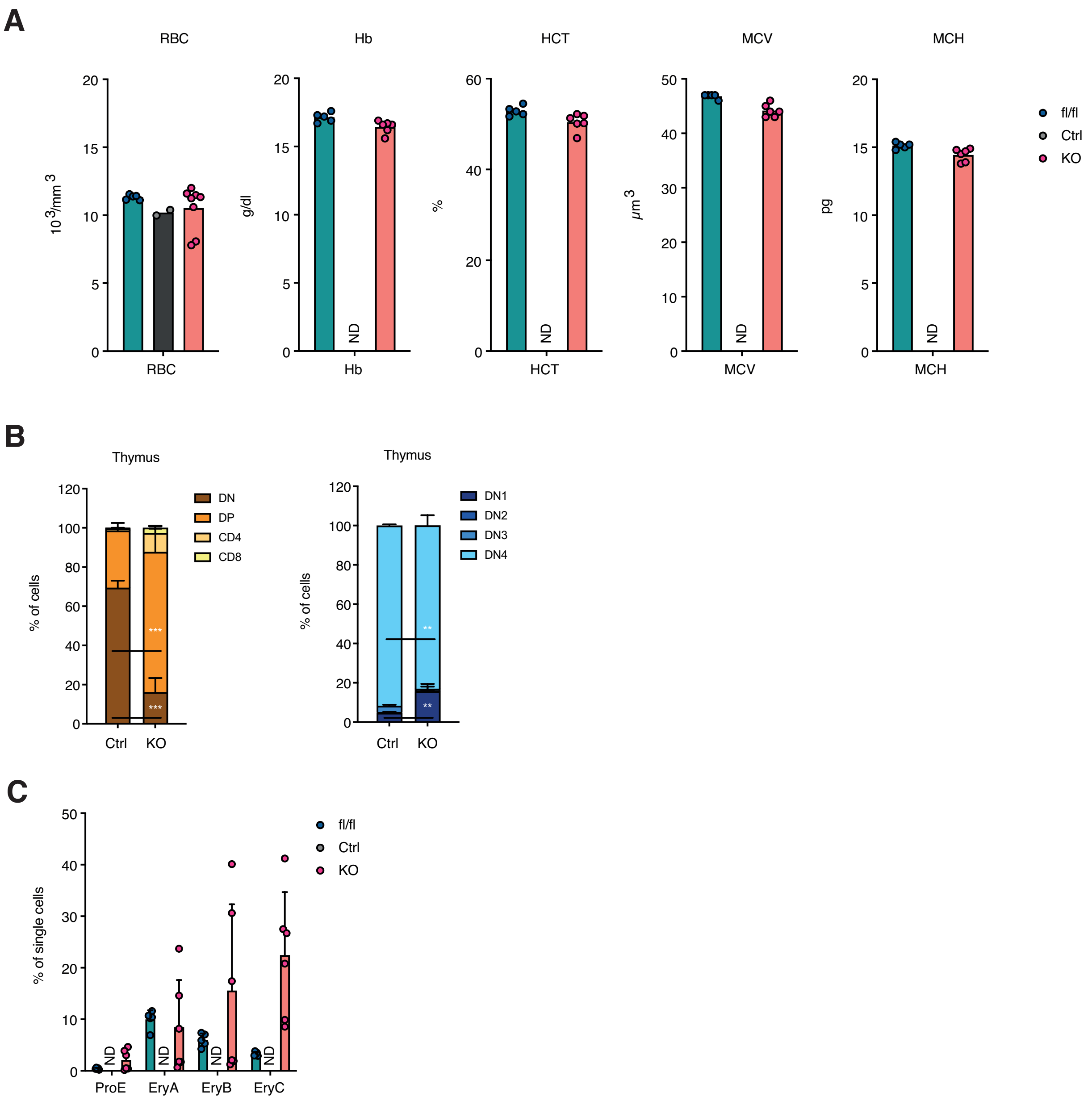

Supplementary Figure 3: Characterization of MxCre *Pabpn1*$^{flox/flox}$ mice. (A) Hemavet analysis of peripheral blood. (B) Flow cytometry-based analysis of the Thymus. (C) Analysis of erythroid maturation in the BM. For all experiments: n = 2-8. n indicates number of biological replicates; 1-3 independent experiments; mean +SD is shown; Two-way ANOVA (% of cells panels); *$p < 0.05$; **$p < 0.01$; ***$p < 0.001$; for statistical analysis KO was compared to Ctrl. ND: not determined.
